# Repeated *Aedes albopictus* bites reshape gut microbiota and repattern inflammatory readouts in a murine colitis model

**DOI:** 10.3389/fmicb.2025.1702365

**Published:** 2025-11-24

**Authors:** Zhiqiang Li, Xiaoyuan Kuang, Ge Shan, Jiahong Wu

**Affiliations:** 1 Guizhou Key Laboratory of Microbio and Infectious Disease Prevention and Control, Guizhou Medical University, Guiyang, China; 2 Department of Immunology, College of Basic Medicine, Guizhou Medical University, Guiyang, China; 3 Department of Medical Biochemistry and Microbiology, Uppsala University, Uppsala, Sweden; 4 Liupanshui Center for Disease Control and Prevention, Liupanshui, China

**Keywords:** *Aedes albopictus* bites, press disturbance, gut microbiota, colitis, reshape

## Abstract

The gut microbiota represents a stable and dynamic symbiotic community that maintains host health and shapes immune homeostasis. Environmental exposures can disturb this symbiosis, yet the impact of repeated vector biting on host microbial communities has not been explored. Here, we investigated how repeated *Aedes albopictus* mosquito bites influence gut microbiota composition and stability in a murine model of dextran sulfate sodium (DSS)–induced colitis. Mice were repeatedly exposed to mosquito bites over several weeks prior to DSS treatment, and fecal microbiota were profiled using 16S rRNA sequencing at baseline, during inflammation (day 7), and recovery (day 14). Mosquito biting acted as a “press disturbance,” increasing microbial richness and community dispersion at baseline compared to unbitten controls. During DSS challenge, mosquito-exposed mice exhibited distinct microbial trajectories relative to DSS-only mice, including altered relative abundance of taxa such as *Lactobacillus*. These microbiota shifts were associated with changes in host inflammatory readouts, including elevated IL-6 during induction and partial normalization by day 14, as well as modest hematological adjustments. Our findings demonstrate that repeated vector exposure can reshape the gut microbiota, modulating the stability and composition of this core host symbiosis under inflammatory stress. These results highlight the sensitivity of symbiotic microbial communities to ecological perturbations and suggest that vector–host interactions may represent an underappreciated factor influencing host–microbe partnerships.

## 1 Introduction

Blood-feeding arthropods, including mosquitoes, initiate complex interactions with vertebrate hosts through the injection of saliva during probing and feeding. Mosquito saliva is composed of pharmacologically active molecules that facilitate feeding and evade host defense responses, including anti-coagulants, vasodilators, and immunomodulatory proteins (Guo et al., 2025; Vander Does et al., 2022). These salivary components can trigger both local and systemic immune activation, including immediate hypersensitivity responses via IgE-mediated mast cell degranulation and delayed-type hypersensitivity involving T-cell recruitment and cytokine release (Vander Does et al., 2022; Arnoldi et al., 2023). While such immune responses have been well-characterized in the context of pathogen transmission, the physiological consequences of repeated, non-infectious mosquito exposure remain underexplored.

*Aedes albopictus*, a globally invasive mosquito species, is an ecologically relevant model for studying such interactions. Its global range continues to expand due to climate change and land-use modification, bringing it into frequent contact with both humans and wildlife (Kraemer et al., 2015; Ryan et al., 2019). Unlike many mosquito species with narrow host preferences, *A. albopictus* is an opportunistic generalist biter, making repeated exposure a common occurrence in many environments. These non-infectious biting events constitute an underrecognized but persistent ecological pressure, independent of vector-borne disease transmission.

Host-associated microbial communities, particularly in the gastrointestinal tract, play essential roles in maintaining immune homeostasis, nutrient absorption, and epithelial barrier integrity (Lozupone et al., 2012; David et al., 2014). These communities are dynamic and responsive to a variety of internal and external perturbations. For example, antibiotics, dietary shifts, and inflammatory injury have all been shown to restructure microbial diversity, reduce compositional stability, and induce transitions between community states (Miao et al., 2025; Rooks and Garrett, 2016). However, the potential role of vector biting as an ecological modifier of gut microbial structure has not been directly tested. This is surprising, given that skin–gut communication is increasingly recognized as a biologically relevant pathway in health and disease.

Evidence supporting a skin–gut axis suggests that cutaneous immune events, such as inflammation, injury, or allergic responses, can influence gut immune tone, epithelial function, and microbial community composition (Mahmud et al., 2022; De Pessemier et al., 2021). Experimental studies have shown that skin injury or inflammation can modulate gut mast cell populations, increase intestinal permeability, or alter mucosal immune signaling (Leyva-Castillo et al., 2019; Dokoshi et al., 2021). These effects are thought to be mediated by circulating cytokines, neuropeptides, and systemic immune activation. Importantly, human epidemiological data show that individuals with chronic inflammatory skin diseases such as atopic dermatitis and psoriasis have a higher incidence of inflammatory bowel diseases, further supporting the idea of cross-barrier immune communication (de Lusignan et al., 2022; Wang H. et al., 2025).

There is increasing interest in how vector–host interactions affect host physiology beyond pathogen transmission. In the case of malaria, host gut microbiota composition influences susceptibility to *Plasmodium* infection, while in mosquitoes, gut-resident bacteria impact vector competence for viral and protozoan pathogens (Sriboonvorakul et al., 2023; Mandal and Schmidt, 2023; Villarino et al., 2016). This bidirectional interaction has shaped efforts to manipulate vector microbiomes for disease control. While recent studies have reported that ectoparasitic infestations such as those caused by ticks or fleas can modulate gut microbiota composition in rodents (Wang et al., 2024; Wang Z. et al., 2025; Pandian, 2023). In the absence of infection, when mosquitoes probe and feed on blood, the host skin experiences not only mechanical stimulation from mosquito mouthparts but also systemic immune responses characterized by altered cytokine levels and changes in innate and adaptive immune cell populations in the skin, blood, spleen, and bone marrow (Vogt et al., 2018; Guerrero et al., 2022). Whether these immune alterations, through the skin–gut axis, could influence gastrointestinal homeostasis or potentially affect the development of gut-associated diseases remains an open and underinvestigated question.

In this study, we used a murine model to test the hypothesis that repeated *Aedes albopictus* biting, even in the absence of pathogens, functions as a low-grade ecological disturbance capable of modulating gut microbial community structure. Because mosquito saliva contains immunomodulatory molecules that can elicit both local and systemic immune activation, repeated biting may alter systemic inflammatory tone and mucosal immune balance. Such systemic immune effects have been linked to gut barrier function and microbiota composition through the skin–gut axis. We therefore further investigated whether this exposure history alters microbiome trajectories or inflammatory phenotypes following an acute colitis challenge induced by dextran sulfate sodium (DSS), which serves as a pulse disturbance. By combining 16S rRNA gene sequencing with inflammatory readouts (IL-6 expression, MPO level, NE activity and colon length), we assess how mosquito exposure history influences both microbial ecology and host physiology during acute inflammation and recovery. In doing so, we expand the conceptual scope of vector–host interactions, highlighting microbiome-mediated consequences of non-infectious arthropod exposure on vertebrate health.

## 2 Materials and methods

### 2.1 Mice and mosquitos

BALb/c mice that were littermates and were kept in the Department of Immunology, College of Basic Medicine, Guizhou Medical University. All mice were treated humanely and housed in an enriched environment. Permission (No. 2100005) for experiments with mice was granted by Guizhou Medical University's Experimental Animal Center. Female *Aedes albopictus* mosquitoes were obtained from a laboratory colony maintained under controlled conditions (27 ± 1 °C, 14:10 h light:dark cycle, without exposure to pathogens) at the mosquito room of Guizhou Key Laboratory of Microbio and Infectious Disease Prevention and Control, Guizhou Medical University.

### 2.2 Experimental mouse model

Littermate BALB/c mice (10–12 weeks old) were divided into four groups: Kong (blank control), Bite, DSS, and Bite + DSS. Each group contained at least six mice (*n* ≥ 6). For the Bite treatment, mice were sensitized to female Aedes albopictus bites once per week for four consecutive weeks (on experimental days −28, −21, −14, and −7) before DSS exposure, totaling four biting sessions. Each session lasted until all mosquitoes had completed blood feeding. Prior to each biting session, mice were anesthetized by intraperitoneal injection of ketamine (75 mg/kg) and xylazine (10 mg/kg) to ensure immobility and minimize discomfort. Anesthetized mice were placed in mouse restrainers and exposed to 50 female *A. albopictus* mosquitoes which were starved for 24 h, inside an 8,000 cm3 cotton net cage. Mice were immobilized until all mosquitoes completed blood feeding.

To experimentally induce colitis using DSS, mice in the DSS and Bite + DSS groups were provided *ad libitum* access to 4% DSS (36,000–50,000 Da, 0216011050, MP Bio) in drinking water for 7 days, followed by *ad libitum* access to sterile water for 7 days. Body weight was recorded daily and the weight changes of groups were evaluated. Fresh fecal samples were collected on days 0, 7, and 14. On day 7 or 14, mice were euthanized using CO_2_ inhalation followed by cervical dislocation, in accordance with institutional animal care and use protocols. Blood samples were collected for hematological analysis, and colon tissues were harvested for histological examination, gene expression analysis, and protein quantification to assess inflammation and disease progression.

### 2.3 Colon sampling and morphological assessment

In brief, 1 cm of the collected colon was fixed with 4% formalin overnight then embedded in paraffin and cut into 4–5 mm tissue sections. The sections were mounted on slides and stained with hematoxylin and eosin (H&E). Pathological changes were assessed by light microscopy.

### 2.4 IL-6 and myeloperoxidase (MPO) detection by ELISA

In brief, the colon tissues were stored in liquid nitrogen and smashed into tissue powder, of which 50 mg was suspended in Hank's balanced salt solution and then spun down by 15,000 × *g* centrifugation for a half hour at 4 °C to an insoluble pellet. Using the manufacturer's protocol, the colonic concentrations of IL-6 and MPO in the obtained supernatant were determined using mouse IL-6 (R&D, #DY406-05) and MPO (Cat. No. ab275109, Abcam) ELISA developmental kits.

### 2.5 Assays for determining colonic neutrophil elastase (NE) activity

Protocols were performed as described in Li et al. (2020). To assess NE activity, 20 μl of 10 μM elastase substrate (#L-1770, Bachem) was incubated with 50 μl of the obtained colonic tissue supernatant in 130 μl of the NE reaction buffer (MilliQ water, 100 mM Tris-HCl, 150 mM NaCl, 0.05% Tween-20, 0.1% BSA; pH = 8.5). The absorbance (optical density, OD) at 405 nm was determined at hour 0 and the next day, and the difference was calculated. Trypsin-like and chymotrypsin-like activities are expressed as ΔOD and the NE activity as milli-ΔOD per minute per mg of tissue.

### 2.6 16S rRNA gene sequencing and microbiome analysis

The protocol was performed as described in Li Z. et al. (2025). Total DNA was isolated from the fecal samples collected from each mouse on days 0, 7, and 14 (corresponding to baseline, acute colitis, and recovery phases, respectively) (*n* ≥ 4 per group). Fecal material was chosen because it provides a representative snapshot of the luminal microbial community of the distal colon, the primary site of inflammation in the DSS model. Using fecal samples allowed non-invasive, longitudinal collection from the same animals, minimizing inter-individual variation and animal sacrifice while remaining comparable to established colitis microbiome studies. Approximately 200 mg of freshly collected fecal material per individual was used for DNA extraction using the DNeasy^®^ PowerSoil^®^ Pro Kit (QIAGEN, USA) according to the supplier's protocol. The 16S rRNA V3–V4 hypervariable regions of fecal and colonic bacteria were amplified with the extracted genomic DNA using primers 338 Forward (5′-ACT CCT ACG GGA GGC AGC AG-3′) and 806 Reverse (5′-GGA CTA CHV GGG TWT CTA AT-3′), as previously descrbed in Li Z. et al. (2025). The PCR products were examined by gel electrophoresis and purified with the AxyPrep DNA Gel Extraction Kit (Axygen Biosciences, Axygen, USA). Sequence libraries were generated using the NEXTFLEX^®^ Rapid DNA-Seq Kit. Sequencing was performed using the Illumina MiSeq PE300 and NovaSeq PE250 platforms (Illumina, San Diego, USA) by Majorbio (Shanghai, China). The MiSeq platform was used for the initial batch of samples, while the NovaSeq platform was used for subsequent sequencing runs to increase throughput. Data from both platforms were processed using the same bioinformatics pipeline to ensure consistency and comparability across datasets. Quality of the original sequence data was assessed using fastp (https://github.com/OpenGene/fastp, version 0.20.0) and spliced using FLASH (https://github.com/OpenGene/fastp, version 1.2.7). Operational taxonomic units (OTUs) were clustered based on a 97% similarity threshold by UPARSE (version 7.1). The analysis of taxonomy for each sequence was performed by comparing the Ribosomal Database Project (RDP) Classifier against the 16S rRNA database. The α-diversity of bacteria was calculated based on the OTUs using the ACE, Chao, Simpson Sobs and/or Shannon indices. Principal coordinates analysis (PCoA) of Bray–Curtis distance was utilized to estimate the β-diversity of the bacterial data. The datasets presented can be found in online repositories (NCBI, PRJNA1062031). The data were analyzed through the free online platform of majorbio cloud platform (cloud.majorbio.com).

### 2.7 RNA extraction and detection of IL-6 expression using qPCR

To extract colonic RNA, colon tissues of the mice for which the weight change was closest to the average of the weight curves were smashed into powder in liquid nitrogen with a mortar and pestle. Then, RNA extraction was performed, with the homogenized tissue powder resuspended in Trizol agent (Thermo Fisher Scientific) according to the manufacturer's instructions. To remove traces of genomic DNA, the extracted RNA was treated with DNase I during the procedure. The 260/280 and 260/230 ratios were measured to assess the quality of the extracted RNA. Using quantitative real-time polymerase chain reaction (qPCR), the relative expression levels of IL-6 were analyzed. The total extracted RNA was reverse-transcribed to cDNA using the Revert Aid H Minus First Strand cDNA Synthesis Kit (Thermo Fisher Scientific) according to the supplier's protocol. Maxima SYBR Green/ROX qPCR Master Mix (Thermo Fisher Scientific) was used for the qPCR, and the expression of GAPDH was used for normalization according to the guidelines of AB Applied Biosystems (Step One Plus Real Time PCR systems). The fold change in gene expression was calculated using the Livak method (2^−ΔΔ*CT*^) as described by Livak and Schmittgen (Livak and Schmittgen, 2001). Primers are indicated in Supplementary Table 1.

### 2.8 Statistical analysis

Statistical analysis of the experimental data between two groups involved a *t*-test with Welch's correction using GraphPad Prism software. Differences in bacteria based on relative abundance at the species or genus level were analyzed using the Student's *t*-test to compare two groups and one-way ANOVA to compare three groups. *P*-values < 0.05 were considered to be statistically significant.

## 3 Results

### 3.1 Repeated mosquito bites altered gut microbial diversity and composition

**Figure 1:**
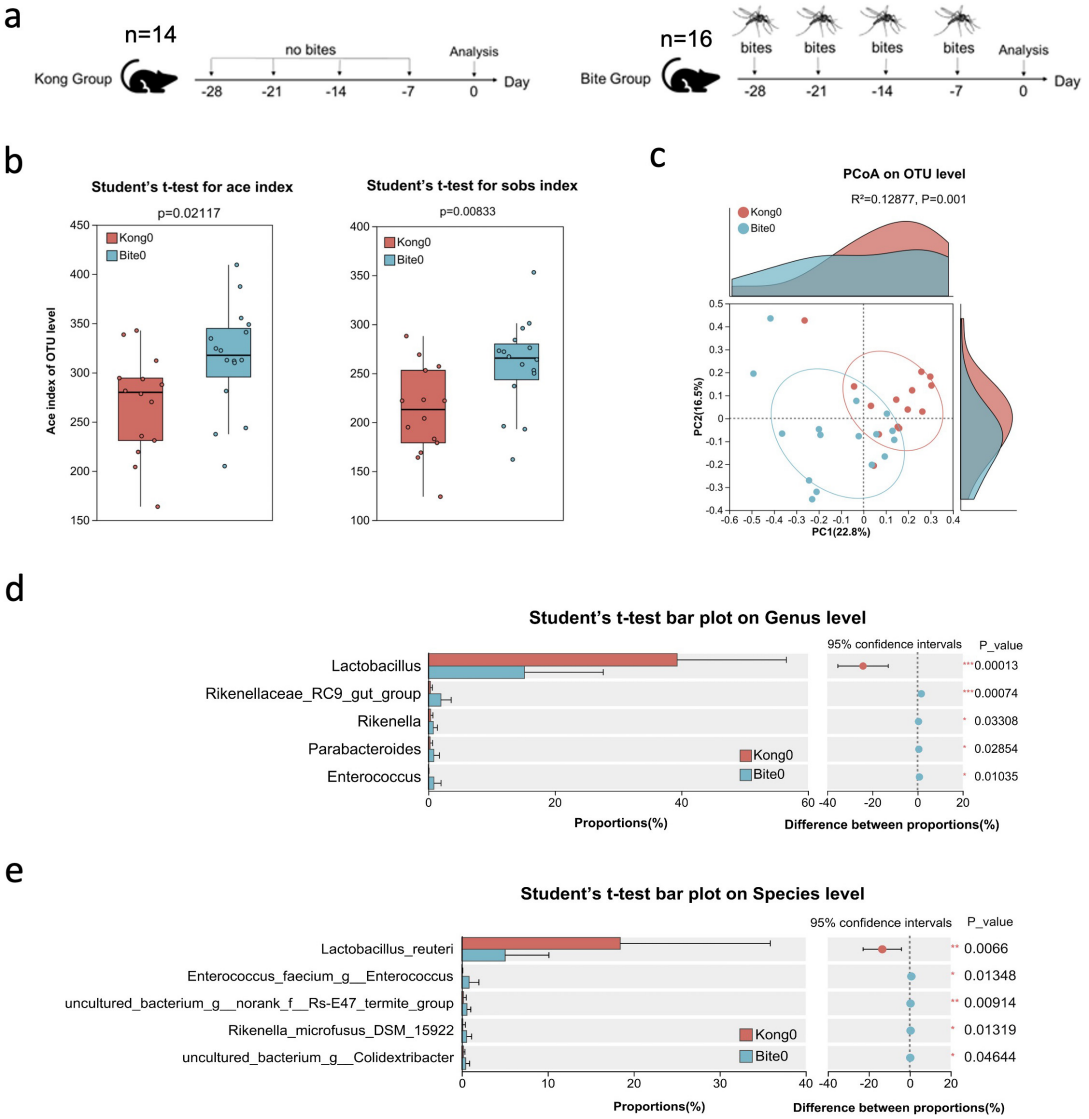
Experimental design and baseline characterization of gut microbiota in control and mosquito bite-exposed mice. **(a)** Schematic of the experimental setup: mice in the Kong group received no mosquito bites, while mice in the Bite group were exposed to *Aedes albopictus* bites on Days −28, −21, −14, and −7. Fecal samples were collected on Day 0 for microbiota analysis. **(b)** Box plots of alpha diversity metrics (ACE and Sobs indices) comparing Kong0 and Bite0 groups. **(c)** Principal Coordinates Analysis (PCoA) plot based on OTU-level Bray–Curtis dissimilarity showing clustering patterns of Kong0 and Bite0 groups. **(d)** Bar plot comparing the relative abundance of dominant bacterial genera between Kong0 and Bite0 groups, along with confidence intervals and *p*-values. **(e)** Bar plot comparing the relative abundance of dominant bacterial species between Kong0 and Bite0 groups, along with confidence intervals and *p*-values. Significant differences are indicated as *P < 0.05, **P < 0.01, ***P < 0.001.

To examine whether repeated *Aedes albopictus* biting influences the gut microbiota, mice were exposed to four times per week for four consecutive weeks (Days −28, −21, −14, and −7 relative to the start of analysis). The Kong (control) group included 14 mice (*n* = 14) and the Bite group included 16 mice (*n* = 16). Fecal samples were collected from individual mice at Day 0 for 16S rRNA gene sequencing to compare microbiota profiles between bite-exposed and control mice (Figure 1a). Alpha diversity metrics showed significantly elevated species richness and diversity in the bite group (Bite0) compared to control (Kong0). Specifically, both the ACE index and observed species (Sobs) index were significantly higher in Bite0 mice (ACE: *p* = 0.021; Sobs: *p* = 0.009) (Figure 1b), indicating an increase in overall microbial richness following mosquito exposure. Principal coordinates analysis (PCoA) based on OTU-level Bray–Curtis distances revealed distinct clustering between groups (*R*^2^ = 0.12877, *p* = 0.001), suggesting that repeated mosquito bites led to a shift in microbial community structure (Figure 1c). Differential abundance analysis at the genus level identified significantly decreased proportions of *Lactobacillus* in bite exposed mice, while *Rikenellaceae_RC9_gut_group*, and *Rikenella, Parabacteroides* and *Enterococcus* were elevated compared with control (Figure 1d). Among the identified species, *Lactobacillus reuteri* was significantly more abundant in the Kong0 group, while *Enterococcus faecium, uncultured bacterium g__norank f__Rs-E47 termite group, Rikenella microfusus DSM 15922*, and *uncultured bacterium g__Colidextribacter* were more abundant in the Bite0 group (Figure 1e). These findings indicate that repeated, pathogen-free mosquito biting is associated with distinct baseline differences in gut microbiota composition and diversity. While these observations do not establish a causal relationship, they suggest that prior mosquito exposure may influence host microbial ecology in ways that could potentially affect subsequent inflammatory responses.

### 3.2 Repeated mosquito biting induced progressive alterations in gut microbial composition

**Figure 2:**
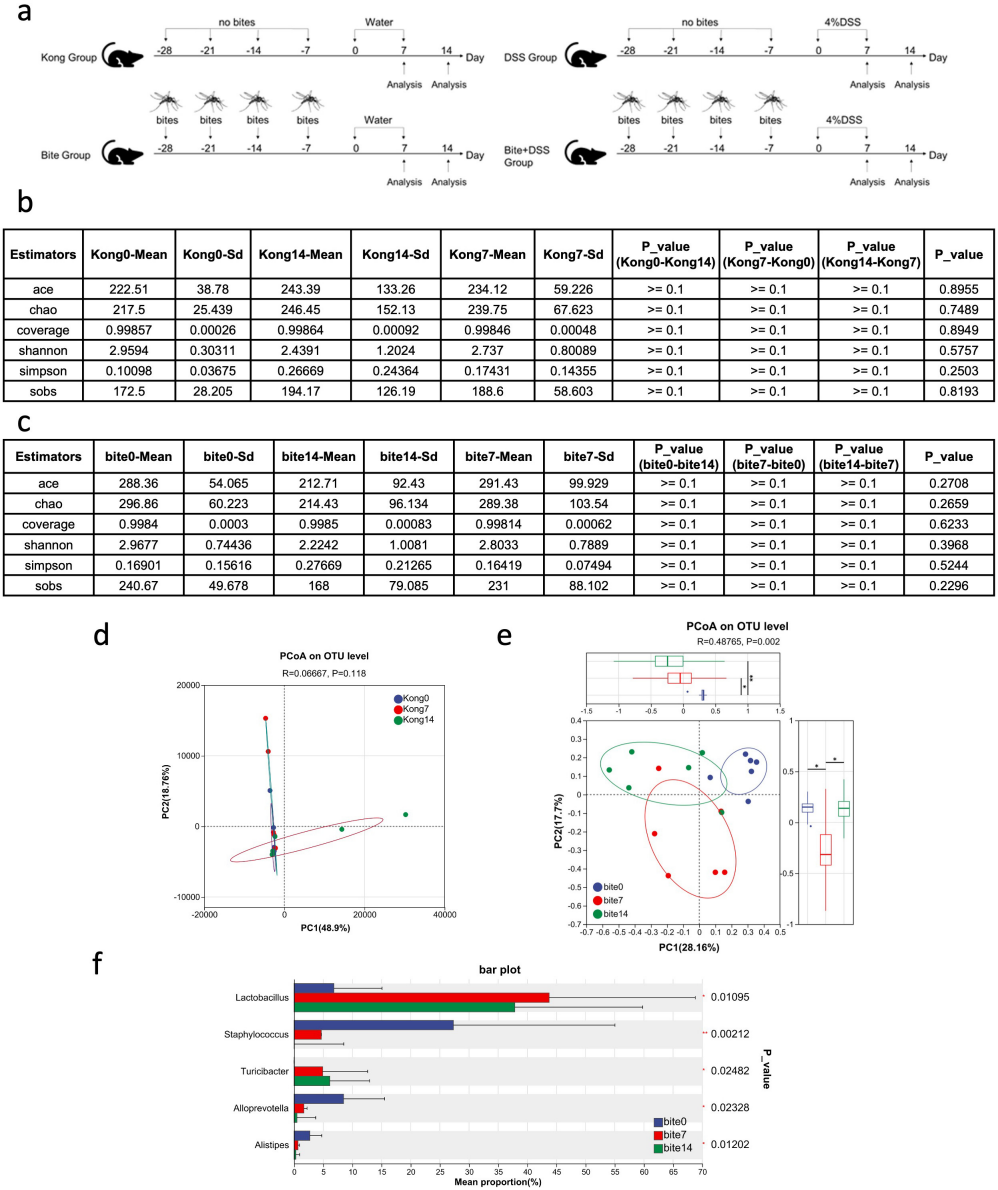
Experimental timeline and analysis of gut microbiota composition across control and mosquito bite groups over time. **(a)** Schematic diagram of the experimental design showing fecal sample collection on Days 0, 7, and 14 in Kong and Bite groups, as well as subsequent DSS treatment groups. **(b)** Table summarizing alpha diversity indices (ACE, Chao1, Coverage, Shannon, Simpson, and Sobs) and corresponding statistical comparisons across time points in the Kong group. **(c)** Table of alpha diversity indices and comparisons across Days 0, 7, and 14 in the Bite group. **(d)** Principal Coordinates Analysis (PCoA) plot based on OTU-level Bray–Curtis distances for the Kong group at Days 0, 7, and 14. **(e)** PCoA plot based on OTU-level Bray–Curtis distances for the Bite group at Days 0, 7, and 14, with box plots and statistical comparisons of group dispersion. **(f)** Bar plot showing the relative abundance of major bacterial genera in the Bite group at Days 0, 7, and 14, with associated *p*-values for differences in mean proportions. Significant differences are indicated as *P < 0.05, **P < 0.01.

To investigate how prior mosquito exposure affects gut microbiota dynamics during colitis progression and recovery in mice, we collected fecal samples from control and bite-exposed mice at three timepoints: before DSS treatment (day 0), after acute colitis (day 7), and post-recovery (day 14) (Figure 2a). We firstly determined whether gut microbiota composition varied spontaneously over time in the absence of mosquito exposure, we profiled the fecal microbial communities of unbitten Kong mice at days 0, 7, and 14. Alpha diversity metrics (ACE, Chao1, Shannon, Simpson, and Sobs indices) remained consistent across all three timepoints (Figure 2b), with no statistically significant differences observed (*p* ≥ 0.1 for all pairwise comparisons). PcoA showed no significant clustering by timepoint in Kong mice (*R*^2^ = 0.0667, *p* = 0.118; Figure 2d), indicating that overall microbial community composition remained largely unchanged during the 14-day period. These results suggest that microbial richness and evenness were stable over time in the control condition. Beta diversity analysis based on Bray–Curtis dissimilarity further supported microbiota stability.

Then we evaluated whether repeated *Aedes albopictus* exposure modulated gut microbial community dynamics over time, we analyzed fecal microbiota profiles from bite-exposed mice at days 0, 7, and 14 (Bite0, Bite7, Bite14). Alpha diversity metrics (ACE, Chao1, Shannon, Simpson, and Sobs) remained statistically unchanged across timepoints (Figure 2c), indicating that repeated mosquito exposure did not significantly alter species richness or overall evenness. This suggests that the biting regimen influenced community structure rather than total diversity. Beta diversity analysis using Bray–Curtis dissimilarity revealed clear time-dependent shifts in microbial composition. PCoA showed distinct clustering of microbial communities by timepoint (*R*^2^ = 0.4785, *p* = 0.002; Figure 2e), with increasing divergence observed between Bite0 and Bite14 samples. This trajectory separation was not present in unbitten controls (Figure 2d), supporting a bite-specific effect. Taxonomic analysis revealed substantial shifts in relative abundance among key microbial genera (Figure 2f). The relative abundance of *Lactobacillus* and *Turicibacter* increased progressively from day 0 to day 7 and 14. In contrast, *Staphylococcus, Alloprevotella*, and *Alistipes* were significantly reduced.

These results demonstrate that repeated mosquito biting can act as a press disturbance, driving gradual reorganization of gut microbial communities. The absence of these shifts in unbitten controls further supports a causal relationship between vector exposure and microbiota restructuring, even in the absence of overt colitis or infection.

### 3.3 Repeated mosquito exposure restructured the gut microbiota during colitis induction and recovery

Next we investigated whether repeated exposure to mosquito bites alters the gut microbiota in the context of intestinal inflammation.

**Figure 3:**
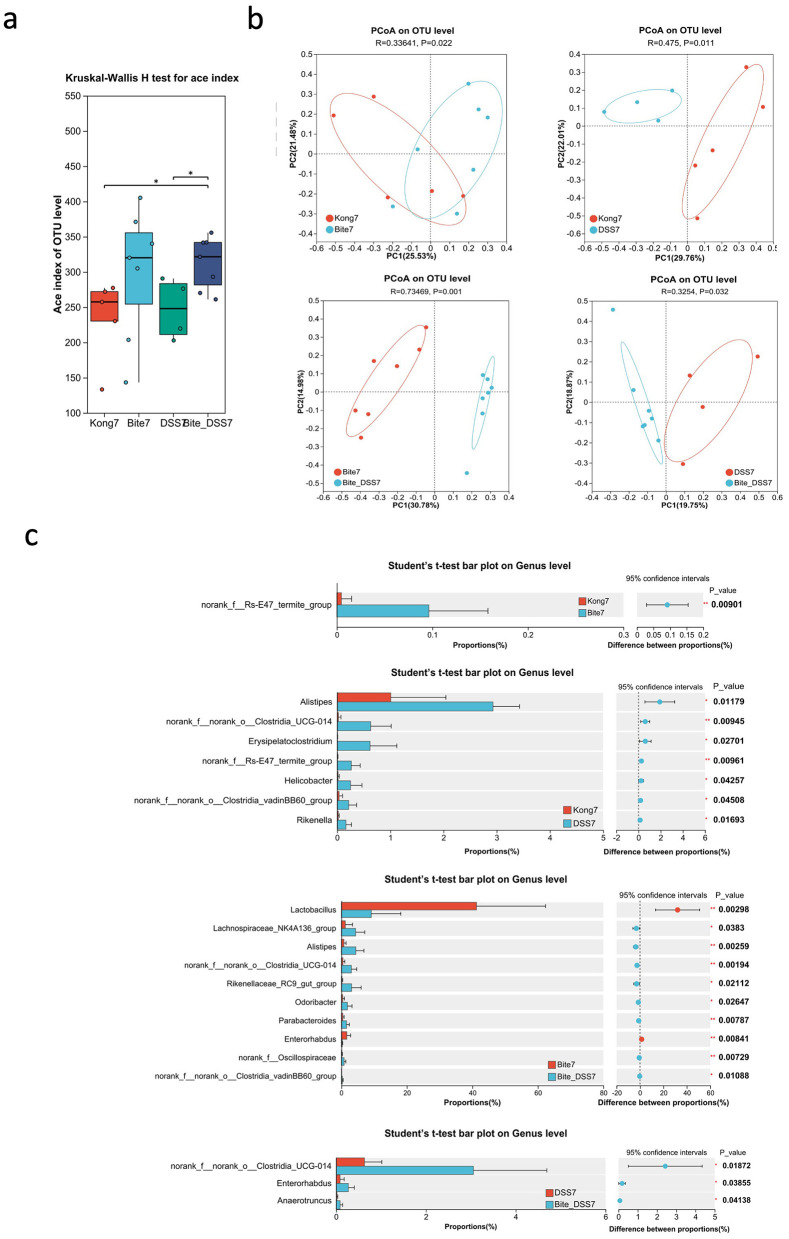
Microbial diversity and taxonomic composition of fecal microbiota at Day 7 following mosquito exposure and/or DSS treatment. **(a)** Box plot of ACE index comparing gut microbial alpha diversity across experimental groups (Kong7, Bite7, DSS7, and Bite_DSS7). **(b)** Principal Coordinates Analysis (PCoA) plots based on OTU-level Bray–Curtis distances showing pairwise comparisons of microbiota structure between Kong7 vs. Bite7, Kong7 vs. DSS7, Bite7 vs. Bite_DSS7, and DSS7 vs. Bite_DSS7. **(c)** Bar plots showing genus-level taxonomic comparisons among Kong7 vs. Bite7 (top left), Kong7 vs. DSS7 (top right), Bite7 vs. Bite_DSS7 **(bottom left)**, and DSS7 vs. Bite_DSS7 (bottom right), including confidence intervals and *p*-values for statistically different genera. Significant differences are indicated as *P < 0.05, **P < 0.01.

By day 7, the Ace index at the OTU level (Figure 3a) revealed significant differences in species richness among the groups. Both Bite7 and Bite_DSS7 showed higher Ace indices compared with DSS7 (*P* < 0.05), suggesting that bite exposure increased microbial richness, while DSS treatment alone reduced it. The combined Bite + DSS7 group maintained relatively high richness, indicating a partial restoration of microbial diversity under combined treatment conditions. PCoA based on OTU-level Bray–Curtis distances demonstrated distinct clustering among the groups (Figure 3b). The microbial community structure of Bite7 was significantly separated from Kong7 (*R* = 0.336, *P* = 0.022), indicating that bite exposure alone altered gut community composition. Similarly, DSS7 differed significantly from Kong7 (*R* = 0.475, *P* = 0.011). A pronounced separation was observed between Bite7 and Bite + DSS7 (*R* = 0.735, *P* = 0.001), suggesting that DSS treatment further reshaped the bite-associated microbiota. Finally, DSS7 and Bite + DSS7 also differed significantly (*R* = 0.325, *P* = 0.032), indicating that the combined treatment induced a distinct microbial profile compared with DSS alone.

Comparative analysis of bacterial taxa at the genus level identified several taxa with significantly different relative abundances among the treatment groups (Figure 3c). In the comparison between Kong7 and Bite7, *norank_f__Rs-E47_termite_group* was significantly enriched in Bite7. Between Kong7 and DSS7, *Alistipes, Erysipelatoclostridium, Helicobacter, Rikenella*, and several *Clostridia* members were significantly increased in DSS7. In Bite7 vs. Bite + DSS7, nine genera, including *Lactobacillus, Alistipes, Rikenellaceae_RC9_gut_group, norank_f__Oscillospiraceae*, and *Parabacteroides*, differed significantly. Notably, *Lactobacillus* was markedly reduced in Bite + DSS7. In the DSS7 vs. Bite + DSS7 comparison, *norank_f__norank_o__Clostridia_UCG-014, Enterorhabdus*, and *Anaeroruncus* were more abundant in Bite + DSS7.

**Figure 4:**
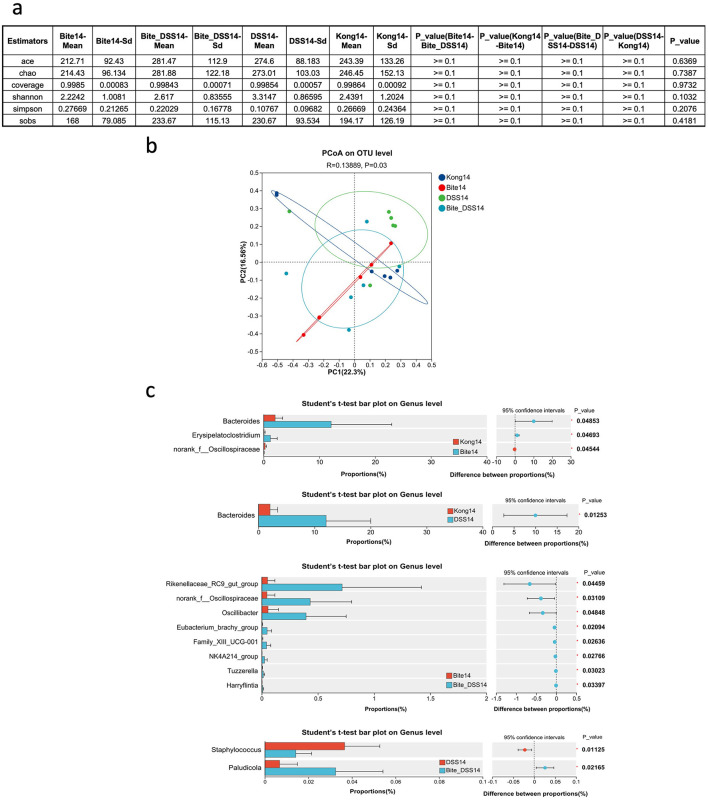
Microbial diversity and taxonomic composition of fecal microbiota at Day 14 following mosquito exposure and/or DSS treatment. **(a)** Summary table of alpha diversity metrics including ACE, Chao1, coverage, Shannon, Simpson, and SOBS indices across experimental groups at Day 14. **(b)** Principal Coordinates Analysis (PCoA) plot based on OTU-level Bray–Curtis distances comparing microbial community structure among Kong14, Bite14, DSS14, and Bite_DSS14 groups. **(c)** Bar plots displaying genus-level taxonomic differences between Kong14 vs. Bite14 (top panel), Kong14 vs. DSS14 (middle-top), Bite14 vs. Bite_DSS14 (middle-bottom), and DSS14 vs. Bite_DSS14 **(bottom)**, with 95% confidence intervals and *p*-values for genera showing statistically significant differences. Significant differences are indicated as *P < 0.05.

By Day 14, alpha diversity metrics across all groups, including Kong14, DSS14, Bite14, and Bite + DSS14 showed no significant differences (Figure 4a), indicating recovery of species richness and evenness regardless of prior exposure history. PcoA based on OTU-level data revealed clear clustering patterns among the four groups. The first two principal coordinates explained 22.3% (PC1) and 16.5% (PC2) of the total variance, respectively. Samples from the four groups showed partial overlap. A ANOSIM test showed a statistically significant difference in microbial community composition among the groups (*R* = 0.13889, *P* = 0.03; Figure 4b), suggesting that treatment effects modestly influenced the gut microbiota structure.

Comparative analysis of gut microbial composition at the genus level revealed several taxa with significantly different relative abundances among the experimental groups (Figure 4c). In the comparison between Kong14 and Bite14, *Bacteroides, Erysipelatoclostridium*, and *norank_f__Oscillospiraceae* were significantly altered. *Bacteroides* showed higher abundance in Bite14, while *Erysipelatoclostridium* and *norank_f__Oscillospiraceae* were relatively more enriched in Kong14. When comparing Kong14 and DSS14, *Bacteroides* was significantly elevated in DSS14, suggesting that DSS treatment promoted its proliferation relative to controls. In the comparison between Bite14 and Bite + DSS14, eight taxa differed significantly, including *Rikenellaceae_RC9_gut_group, norank_f__Oscillospiraceae, Oscillibacter, Eubacterium_brachy_group, Family_XIII_UCG-001, NK4A214_group, Tuzzerella*, and *Harryflintia*. Most of these taxa, particularly *Rikenellaceae_RC9_gut_group* and *Oscillibacter*, were more abundant in the Bite + DSS14 group. Finally, comparison between DSS14 and Bite + DSS14 identified *Staphylococcus* and *Paludicola* as significantly differentially abundant genera. *Staphylococcus* was enriched in DSS14, whereas *Paludicola* showed higher abundance in Bite + DSS14.

Together, these data demonstrate that repeated mosquito biting not only shifts gut microbiota composition prior to colitis, but also modulates microbial responses during and after DSS-induced inflammation. The effects are temporally dynamic, with early increases in diversity giving way to persistent community reconfiguration. The persistence of specific bacterial groups in Bite + DSS14 animals suggests that vector exposure may contribute to long-term alterations in microbial ecology and immune tone, even after resolution of acute colitis. These findings support the hypothesis that vector–host interactions may act as subclinical ecological stressors capable of reprogramming host-associated microbial ecosystems, with implications for host resilience to subsequent environmental or immune perturbations.

### 3.4 Hematological profiling of mice exposed to repeated mosquito bites and DSS-induced colitis

**Figure 5:**
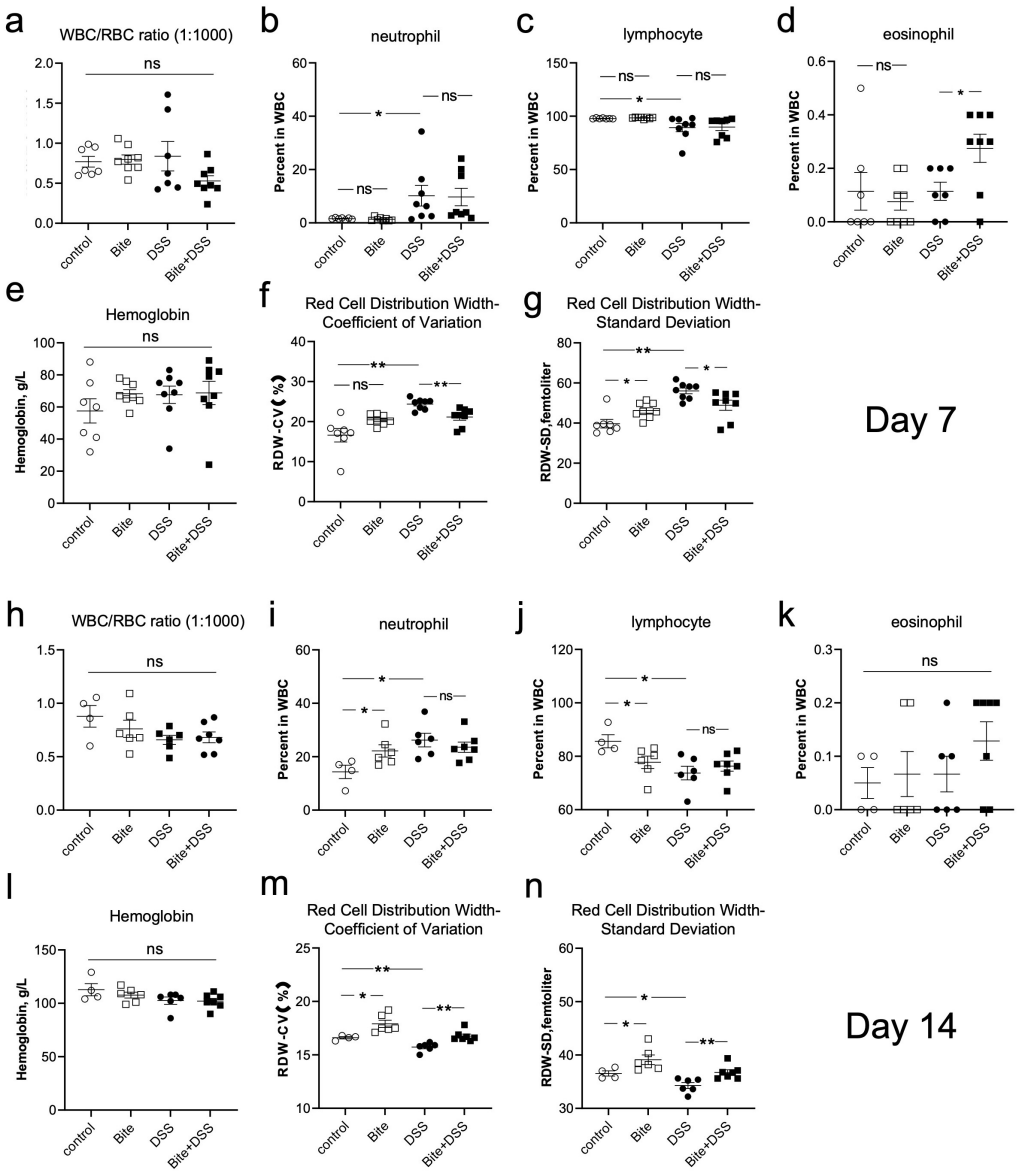
Hematological parameters in mice following repeated Aedes albopictus bites and/or DSS-induced colitis. Hematological analyses conducted on Day 7 post-DSS administration. **(a)** White blood cell (WBC) to red blood cell (RBC) ratio (1:1000). **(b–d)** Percentages of neutrophils, lymphocytes, and eosinophils in WBCs. **(e)** Hemoglobin concentration (g/L). **(f, g)** Red cell distribution width (RDW) presented as coefficient of variation (CV) and standard deviation (SD). **(h–n)** Corresponding hematological parameters measured on Day 14, presented in the same order. Data are shown as mean ± SE and statistical analysis conducted by Welch's *t* test with significant difference presented as *P < 0.05, **P < 0.01.

To investigate systemic hematological responses to colitis and how these might be modulated by repeated *Aedes albopictus* bites, we performed routine blood parameter analyses at both acute (Day 7) and recovery (Day 14) phases of DSS-induced colitis. At Day 7, the WBC/RBC ratio showed no significant differences among groups (Figure 5a). However, compared with the Control group, DSS-treated mice exhibited a marked increase in neutrophil percentage (Figure 5b), accompanied by a decrease in lymphocyte proportion (Figure 5c), indicating an acute inflammatory response. In contrast, eosinophil levels were significantly elevated in the Bite + DSS group compared with DSS alone (Figure 5d), suggesting a mild modulation of immune activation by bite exposure. Hemoglobin concentrations remained stable across all groups (Figure 5e), indicating preserved erythropoiesis. In contrast, red cell distribution width (RDW), both in coefficient of variation (RDW-CV) and standard deviation (RDW-SD), was significantly increased in the DSS group compared with Control, reflecting greater variability in erythrocyte size (Figures 5f, g). Interestingly, RDW values in the Bite + DSS group were significantly lower than in DSS alone, suggesting that bite exposure partially alleviated DSS-induced anisocytosis. The Bite group alone showed minimal hematological alteration relative to controls.

At day 14, the WBC/RBC ratio did not differ significantly (Figure 5h). However, both Bite and DSS groups showed elevated neutrophil percentages and reduced lymphocyte levels compared with the Control group (Figures 5i, j), suggesting a mild but sustained inflammatory trend. Eosinophil counts and hemoglobin concentrations remained unchanged among groups (Figures 5k, l). Notably, RDW-CV and RDW-SD levels were higher in the Bite group than in the Control group (Figures 5m, n), indicating enhanced erythrocyte size heterogeneity during prolonged bite exposure. Importantly, the Bite + DSS group exhibited higher RDW-CV and RDW-SD values compared with DSS alone, implying a complex interaction between bite-induced stimulation and DSS-related systemic effects, possibly reflecting altered red blood cell turnover or compensatory hematopoietic responses. Supplementary Figure 1 expands on platelet-related metrics including platelet counts, mean platelet volume (MPV), platelet distribution width (PDW), and procalcitonin (PCT), a biomarker for systemic inflammation. No significant differences were observed among groups at either Day 7 or Day 14 across these indices.

These findings demonstrate that DSS treatment induced characteristic inflammatory and hematological alterations, including neutrophilia, lymphopenia, and increased red blood cell size variability. Bite exposure alone did not cause overt inflammation but was associated with elevated RDW, suggesting adaptive erythropoietic remodeling. Furthermore, bite stimulation in combination with DSS amplified erythrocyte variability, highlighting an interaction between mosquito bite and inflammation that may influence systemic hematological homeostasis.

### 3.5 Mosquito bites modulate colitis severity and inflammatory responses

**Figure 6:**
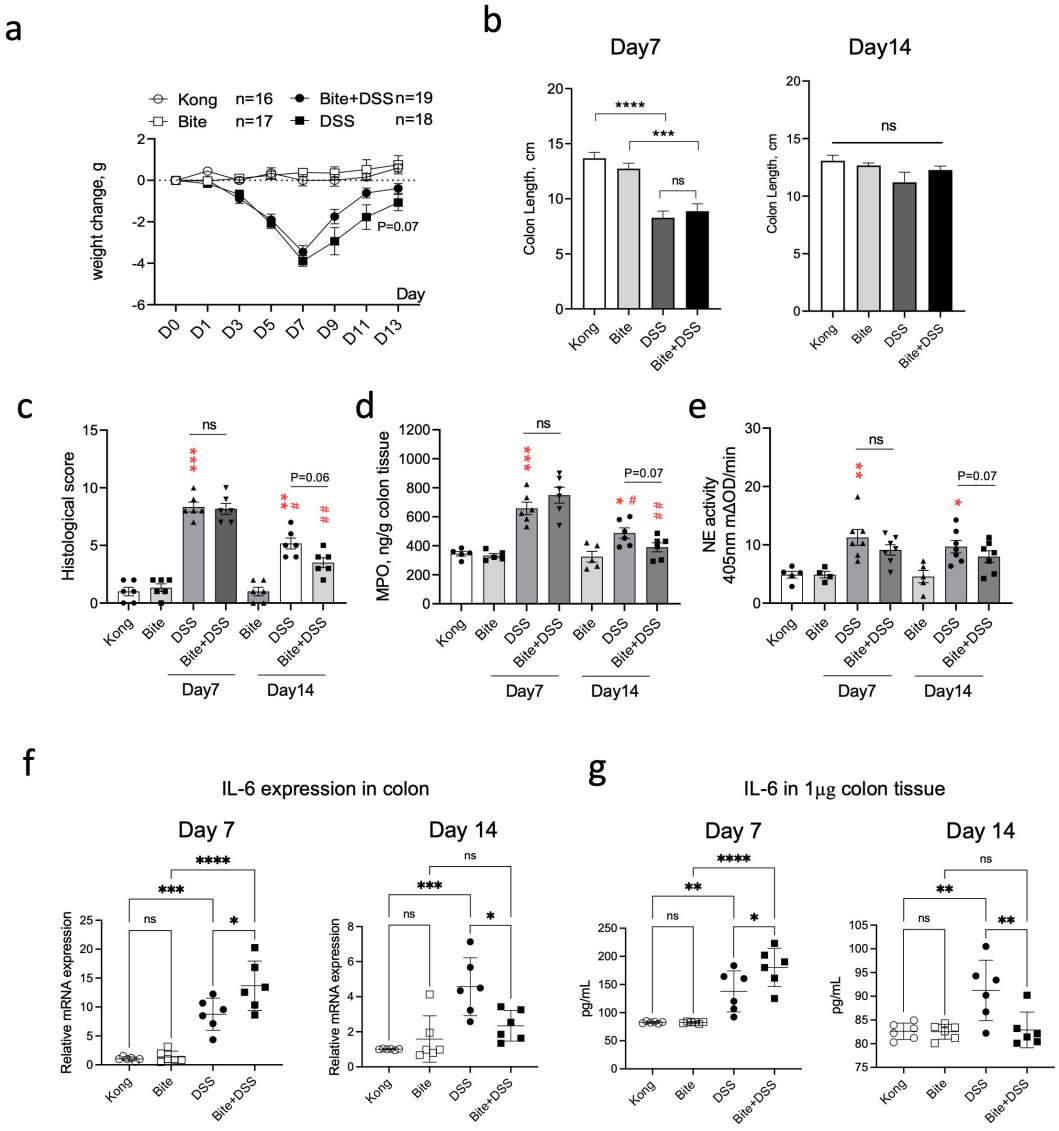
Effects of repeated Aedes albopictus bites on DSS-induced colitis severity and inflammation in mice. **(a)** Body weight changes in mice across treatment groups (Kong, Bite, DSS, Bite + DSS) during the 13-day DSS colitis experiment. **(b)** Colon length measured on day 7 and day 14. **(c)** Histological scores of colon tissue on day 7 and day 14. **(d)** Myeloperoxidase (MPO) concentration in colon tissue. **(e)** Neutrophil elastase (NE) activity in colon tissue. Data are shown as mean ± SE and statistical analysis conducted by Welch's *t* test with significant difference presented as *P < 0.05, **P < 0.01, ***P < 0.001 vs. Kong; ^#^*P* < 0.05, ^##^*P* < 0.01 vs. the same group at day 7; ns, non-significant. **(f)** mRNA expression levels of IL-6 in colonic tissue on day 7 and 14. **(g)** IL-6 protein levels in colon tissue quantified by ELISA on day 7 and 14. Data are shown as mean ± SE and statistical analysis conducted by Welch's *t* test with significant difference presented as *P < 0.05, **P < 0.01, ***P < 0.001, ****P < 0.0001.

To determine whether repeated *Aedes albopictus* bites influence colitis progression and recovery, we monitored clinical and molecular markers in DSS-treated mice with or without prior mosquito exposure. Body weight was reduced across all DSS-treated groups during the acute phase of colitis, with partial recovery observed by day 13 (Figure 6a). Notably, the Bite + DSS group showed a trend toward less weight loss compared to DSS-only mice during recovery (*p* = 0.07), suggesting possible modulation of disease course by bite exposure. Colon length, a marker of colonic inflammation and tissue damage, was significantly reduced in both DSS and Bite + DSS groups at day 7 relative to controls (*p* < 0.0001 and *p* < 0.001, respectively), but no significant difference was observed between the two DSS groups (Figure 6b). By day 14, colon lengths had normalized across groups. Histological analysis of colonic tissues revealed increased inflammatory scores in both DSS and Bite + DSS groups at day 7, with a non-significant trend toward lower histological scores in Bite + DSS mice (*p* = 0.06), which may reflect accelerated tissue recovery (Figure 6c). Neutrophil activity, assessed via myeloperoxidase (MPO) and neutrophil elastase (NE) levels, was elevated in both DSS groups at day 7 (Figures 6d, e). A downward trend in MPO and NE was observed in the Bite + DSS group at day 14 (*p* = 0.07 for both), suggesting altered innate immune activation during resolution. IL-6 mRNA and protein levels in colonic tissues were significantly elevated in DSS mice at day 7, but this increase was attenuated in the Bite + DSS group (Figures 6f, g). At day 14, IL-6 mRNA remained elevated in DSS-only mice but was significantly reduced in Bite + DSS mice (*p* < 0.05), indicating potential immunomodulatory effects of prior mosquito exposure. Together, these findings suggest that repeated mosquito biting prior to colitis induction does not exacerbate acute inflammation but may instead influence recovery by modulating neutrophil activity and IL-6–mediated responses.

## 4 Discussion

This study demonstrates that repeated *Aedes albopictus* mosquito biting (RAB), independent of pathogen transmission, significantly alters gut microbiota composition and modulates systemic immune responses in mice. These alterations were associated with measurable differences in inflammatory markers and hematological parameters during the course of experimental colitis. Our findings expand the concept of vector–host interactions by showing that mosquito exposure alone, without accompanying pathogens, can act as a biologically meaningful environmental pressure with downstream effects on host gut ecology and immune regulation.

*Aedes albopictus*, also known as the “Asian tiger mosquito,” was selected as the biting vector in this study due to its aggressive biting behavior, ecological adaptability, and ongoing global expansion. As one of the most invasive mosquito species worldwide, *A. albopictus* is increasingly establishing itself in temperate regions due to climate change and urbanization (Kraemer et al., 2015). Unlike many mosquito species with narrow host preferences, *A. albopictus* is a generalist biter that readily feeds on humans and wildlife, raising the frequency of non-pathogen-associated biting events in both natural and peri-domestic environments (Bonizzoni et al., 2013; Garrido et al., 2023). Its salivary secretions contain a wide range of pharmacologically active proteins known to provoke strong innate and adaptive immune responses, including mast cell degranulation, eosinophil recruitment, and cytokine release (Vogt et al., 2018; Ribeiro and Francischetti, 2003; Henrique et al., 2019). These immunological properties make *A. albopictus* an ecologically relevant and mechanistically informative model for studying the effects of vector contact beyond pathogen transmission.

To model host–microbiota–vector interactions under ecologically relevant conditions while maintaining experimental control, we used genetically defined laboratory mice housed in naturalistic environments. Unlike specific pathogen free facilities, which exclude environmental microbial exposure and immune stimuli, natural housing systems allow for greater microbiota complexity and more mature immune phenotypes, better reflecting natural populations (Rosshart et al., 2017; Beura et al., 2016). This model enables us to reliably assess the effects of RAB on host physiology and gut ecology, while avoiding the confounding variability associated with wild animals. By combining experimental tractability with environmental realism, our approach provides a robust framework for dissecting how vector exposure can act as an ecological driver of host biology.

Considering that cytokine and chemokine profiles were markedly altered at day 7 following mosquito saliva injection (Vogt et al., 2018), the mosquito-biting regimen was designed to model chronic, low-grade, non-infectious vector exposure rather than an acute insult. Mice were exposed to *Aedes albopictus* bites once per week for four consecutive weeks to simulate the intermittent but sustained contact that typically occurs in endemic regions. This interval allows for repeated immunological stimulation such as mast cell activation, cytokine release, and local inflammation, while providing sufficient time for tissue recovery and gut–immune–microbiota interactions between exposures. Thus, weekly exposure aligns with this immune response window, ensuring that each new bite occurs when the host immune system is still influenced by the prior exposure.

The gut and skin are both complex immune and microbial ecosystems that communicate bidirectionally through neural, endocrine, and immune pathways, forming what is known as the gut–skin axis (Mahmud et al., 2022). Mosquito feeding is far more than a superficial skin event, it triggers a systemic immune dialogue within the host. Mechanical disruption and salivary antigens activate cytokine networks and immune cell redistribution across the skin and bloodstream (Vogt et al., 2018; Guerrero et al., 2022). This broad immunological response could, through the interconnected skin–gut axis, reshape intestinal homeostasis and alter vulnerability to gastrointestinal disease. To explore this possibility, we examined whether RAB affected gut microbial composition, as shifts in bacterial communities can reflect the state of gastrointestinal homeostasis. Although microbial composition varies along the gastrointestinal tract, fecal samples largely reflect the luminal community of the distal colon, which is the principal site of inflammation in DSS-induced colitis. Fecal sampling enables longitudinal analysis of microbial dynamics in the same individuals without repeated invasive procedures, thereby reducing variability and animal use while capturing ecologically relevant changes in gut microbiota composition.

Our Longitudinal 16S rRNA sequencing analysis revealed that RAB was associated with significant alterations in gut microbiota at Day 0 (Figure 1). Among the altered taxa that responded to RAB, the relative abundance of the dominant bacteria *Lactobacillus* was significantly reduced. This genus plays a key role in maintaining epithelial barrier integrity and regulating immune responses, and several *Lactobacillus* strains have been reported to alleviate intestinal inflammation or colitis through immune modulation and barrier protection (Peng et al., 2023; Wang et al., 2023; Jia et al., 2022). Which suggests that RAB exposure is associated with measurable alterations in gut microbiota composition, implying that even non-pathogenic mosquito feeding may exert functional effects on gastrointestinal homeostasis. Previous reports have linked ectoparasite infestations, such as ticks and fleas, to alterations in rodent gut microbiota (Wang et al., 2024; Wang Z. et al., 2025; Pandian, 2023), likely reflecting prolonged immune activation and microbial exchange during chronic parasitism. In contrast, our model isolates the effect of repeated, pathogen-free mosquito biting, which involves brief salivary exposure rather than sustained feeding or infection. This distinction highlights the novelty of our study, which examines how transient, non-parasitic vector contact alone can act as a mild ecological disturbance influencing gut microbial and inflammatory dynamics.

Across the experimental timeline, RAB induced dynamic yet transient alterations in gut microbial composition. By Day 7, no significant difference in α-diversity was detected between the Kong7 and Bite7 groups, although β-diversity remained significantly distinct. At the genus level, only one low-abundance taxon differed between groups, indicating that RAB-induced effects on the microbiota were modest or transient at this stage. By Day 14, there were no significant differences in either α- or β-diversity between the Kong14 and Bite14 groups, suggesting recovery of overall microbial equilibrium. However, the Bite14 group displayed a notable increase in Bacteroides abundance, implying a delayed or selective enrichment of this genus following repeated mosquito exposure. Together, these findings indicate that RAB initially perturbed the gut microbiota but that community composition gradually stabilized over time, with only minor taxonomic shifts persisting at later stages.

Given that gut microbial imbalance, or dysbiosis, is a well-recognized contributor to the initiation and exacerbation of colitis (Joo et al., 2024; Caruso et al., 2020), we employed a dextran sulfate sodium (DSS)-induced colitis model to evaluate the functional relevance of the microbiota changes observed after RAB exposure. In our study, by Day 7, DSS treatment did not significantly alter α-diversity but caused a marked shift in β-diversity. Previous reports have described similar variability, with some showing reduced α-diversity and others finding no change but consistent β-diversity differences (Yang et al., 2023; Wu et al., 2021), likely due to variations in DSS concentration, exposure duration, mouse strain, and housing conditions. By Day 14, there were no significant differences in either α- or β-diversity between the control (Kong14) and DSS-treated (DSS14) groups, indicating partial recovery of microbial diversity and community structure following DSS exposure.

Furthermore, at day 7, bite exposure alone increased microbial richness and partially counteracted the loss of diversity induced by DSS. Enrichment of taxa such as *norank_f__Rs-E47_termite_group* and maintenance of *Lactobacillus* populations in the Bite7 group suggest that immunological or physiological changes associated with bite stimulation may enhance microbial colonization or promote beneficial bacterial growth. In contrast, DSS treatment led to enrichment of *Alistipes, Erysipelatoclostridium, Helicobacter*, and *Rikenella*, genera often associated with mucosal inflammation, epithelial damage, and dysbiosis in colitis models (Parker et al., 2020; Liu et al., 2023; Mueller et al., 2019; Butera et al., 2018). The combined Bite_DSS7 group exhibited both high richness and distinct compositional features, implying that bite exposure modifies the trajectory of DSS-induced microbial disruption, potentially by influencing gut motility, immune activity, or mucosal environment.

By day 14, these alterations became more evident. Bite treatment continued to favor *Bacteroides* enrichment, a genus known for its roles in polysaccharide metabolism and maintenance of gut homeostasis (Zhang et al., 2024; Huang et al., 2024). DSS exposure alone caused a broader restructuring of the microbiota, increasing the abundance of *Rikenellaceae_RC9_gut_group, Oscillibacter*, and *Eubacterium_brachy_group*, taxa frequently linked to chronic inflammation and epithelial stress (Li et al., 2025; Lan et al., 2023; Yan et al., 2022). The Bite + DSS14 group showed the most distinctive microbial profile, characterized by elevated *Rikenellaceae* and *Oscillospiraceae* members and reduced *Lactobacillus* levels, suggesting a synergistic or compounding effect between mosquito-bite-induced immune activation and DSS-mediated epithelial injury on microbial ecology. These findings align with previous studies reporting that combined environmental or chemical stressors can accelerate dysbiosis and delay microbial recovery following intestinal injury.

DSS exposure produced systemic hematological changes typical of acute colitis, including neutrophilia, lymphopenia, and increased red cell distribution width (RDW-CV and RDW-SD). These features are consistent with inflammatory stress and disrupted erythropoiesis, phenomena frequently observed in both experimental colitis and human inflammatory bowel disease (Simeonova et al., 2025; Yang and Merlin, 2024; Carrillo-Palau et al., 2023). Elevated RDW reflects increased heterogeneity in red blood cell size, which can result from cytokine-mediated iron restriction, oxidative stress, or shortened erythrocyte lifespan (Katsaros et al., 2020). In our study, mosquito bite exposure alone produced a mild RDW elevation without significant changes in white-cell counts, suggesting a subclinical hematopoietic adjustment rather than overt inflammation. In combination with DSS, RDW parameters followed a biphasic pattern, initially attenuated during acute inflammation (day 7) but elevated again by day 14. This pattern might indicate that mosquito bite exposure transiently modulated systemic inflammatory tone or erythropoietic activity during recovery. However, given the complexity of hematological regulation and the small effect size, these findings should be interpreted as indicative rather than conclusive of systemic immunomodulation. Mosquito bites have been reported to induce systemic immune responses in some contexts, including mild eosinophilia and Th2-skewed cytokine release (Vogt et al., 2018). Such responses could plausibly alter bone marrow dynamics and cytokine signaling, but confirmation would require longitudinal profiling of hematopoietic and endocrine markers.

The DSS model recapitulates many features of human ulcerative colitis through chemically induced disruption of the epithelial barrier, leading to microbial translocation, immune activation, and mucosal injury. Consistent with prior studies (Simeonova et al., 2025; Yang and Merlin, 2024), DSS administration resulted in clear colonic inflammation in our experiments, evidenced by reduced colon length, histological injury, elevated myeloperoxidase (MPO) and neutrophil elastase (NE) activity, and increased IL-6 expression. Interestingly, mosquito bite exposure alone did not provoke any measurable colonic inflammation or cytokine upregulation. In mice exposed to both mosquito bites and DSS, IL-6 expression and neutrophil activity were slightly lower during the acute phase (day 7), accompanied by a trend toward faster recovery of colon morphology and histology by day 14. These observations could reflect a mild modulatory effect of mosquito saliva on inflammatory signaling. Previous studies have shown that mosquito saliva contains bioactive molecules such as D7 proteins that can suppress local cytokine release and inhibit neutrophil activation (Martin-Martin et al., 2020). While it is tempting to speculate that similar mechanisms may have influenced intestinal responses in our model, the modest magnitude and transient nature of these changes warrant cautious interpretation. It is also possible that systemic physiological stress associated with repeated bite exposure influenced immune responses indirectly, for example through neuroendocrine signaling or glucocorticoid modulation, which have been shown to affect colitis outcomes in other contexts. Further experiments using defined salivary components or controlled stress paradigms would be necessary to disentangle these potential mechanisms.

The interplay between intestinal inflammation, systemic immunity, and external immune stimuli such as mosquito bites is likely multifactorial. DSS-induced barrier disruption promotes neutrophil activation, cytokine release, and microbial translocation (Sun et al., 2024), which can in turn drive hematopoietic stress and alter red blood cell morphology (He et al., 2025). Conversely, peripheral immune activation, such as that induced by mosquito bites, can influence cytokine networks, stress hormone levels through systemic signaling pathways (Guerrero et al., 2022). Our findings suggest that these processes may interact in subtle but measurable ways. The reduced IL-6 expression and partial preservation of microbial diversity in Bite + DSS mice could reflect a transient dampening of systemic inflammation, while later changes in RDW and hematological indices may indicate adaptive hematopoietic compensation. It remains uncertain whether these shifts confer true protection or simply reflect physiological adjustment to multiple simultaneous immune challenges. Importantly, this study does not imply that mosquito bites have a protective or therapeutic effect in colitis; rather, they may represent a biological model for studying peripheral immune modulation and its systemic consequences. Understanding how skin-derived immune signals influence intestinal inflammation could provide new insights into the regulation of mucosal homeostasis and the impact of environmental exposures on gut health.

Our findings align with and expand the concept of the skin–gut axis, a bidirectional communication pathway whereby immune or neuroimmune events at the skin surface can influence distal mucosal sites such as the gut (Mahmud et al., 2022; Jimenez-Sanchez et al., 2025). Previous studies have shown that skin injury or allergen exposure can lead to altered gut immunity and barrier function via cytokine signaling, systemic immune cell recruitment, and microbial changes (Leyva-Castillo et al., 2019; Wiertsema et al., 2021). Here, we show that vector biting, a common environmental exposure in natural ecosystems, can serve as a biologically potent initiator of this axis, with implications for gut inflammation and microbial ecology. Given that mosquito salivary components can elicit both immediate (IgE-mediated) and delayed (type IV) hypersensitivity responses (Vander Does et al., 2022), the systemic immune priming induced by repeated bites may influence both the structure of the gut microbiota and the host's immunological baseline before inflammatory challenges occur.

## 5 Conclusion

In summary, our findings reveal that repeated mosquito biting can reshape host gut microbiota and modulate systemic immune responses, even in the absence of pathogen transmission. These effects have meaningful consequences for intestinal inflammation, as shown in a colitis model, and highlight a previously underappreciated ecological pathway linking arthropod exposure to host health. This work broadens the conceptual framework of vector–host interactions, suggesting that vectors may influence disease outcomes not only by transmitting pathogens, but also by altering host-microbiota-immune system homeostasis through persistent environmental exposure. The ecological relevance of these findings is particularly salient given the rapid global spread of *A. albopictus*. In many environments, vertebrates are exposed to frequent, non-infectious mosquito bites. Our study suggests that these interactions may shape host health trajectories by modulating the immune system and microbial composition in ways not previously recognized. This could be particularly relevant in areas where mosquito exposure is chronic but pathogen load is low, or where host susceptibility to gut inflammation varies. From a translational perspective, our results raise the possibility that environmental arthropod exposure could act as a modifiable factor in inflammatory disease risk, especially in regions with high vector density. Furthermore, they call attention to the need for deeper investigation of the non-pathogenic, ecological roles of vectors, particularly in shaping vertebrate microbial and immune landscapes.

## 6 Limitations of the study

Several limitations should be acknowledged when interpreting the findings of this study. (1) Although the use of laboratory mice housed under naturalistic conditions increases ecological relevance compared to specific-pathogen-free models, the controlled experimental environment still differs from wild settings in terms of microbial exposure, dietary diversity, and environmental stressors. Therefore, extrapolation to wild mammals or human populations should be made with caution. (2)While *Aedes albopictus* was selected due to its expanding geographic range and frequent contact with mammalian hosts, the observed effects may not be generalizable to other mosquito species with differing salivary compositions or biting behaviors. Future studies comparing the immunological and microbiome impacts of different vector species would strengthen ecological inferences. (3) Although we observed correlations between mosquito exposure, gut microbiota shifts, and changes in inflammatory markers, the study does not establish causal mechanisms. Whether specific microbial taxa mediate the immune modulation observed, or vice versa, remains unclear. Metagenomic or metabolomic analyses, along with microbiota transfer experiments, will be necessary to disentangle these relationships. (4) We focused on a limited number of timepoints (day 0, 7, and 14), which may have missed important intermediate dynamics in immune and microbial responses. Higher-resolution temporal sampling would provide a more nuanced view of the trajectory and reversibility of mosquito bite-induced changes.

## Data Availability

The data presented in this study are publicly available. The data can be found at: https://www.ncbi.nlm.nih.gov/bioproject, accession PRJNA1062031.
