# Supplementary figures and images for: Repeated Aedes albopictus bites reshape gut microbiota and repattern inflammatory readouts in a murine colitis model
Source: Front Microbiol. 2025 Nov 24;16:1702365. doi: 10.3389/fmicb.2025.1702365 (PMC12683916; doi:10.3389/fmicb.2025.1702365)

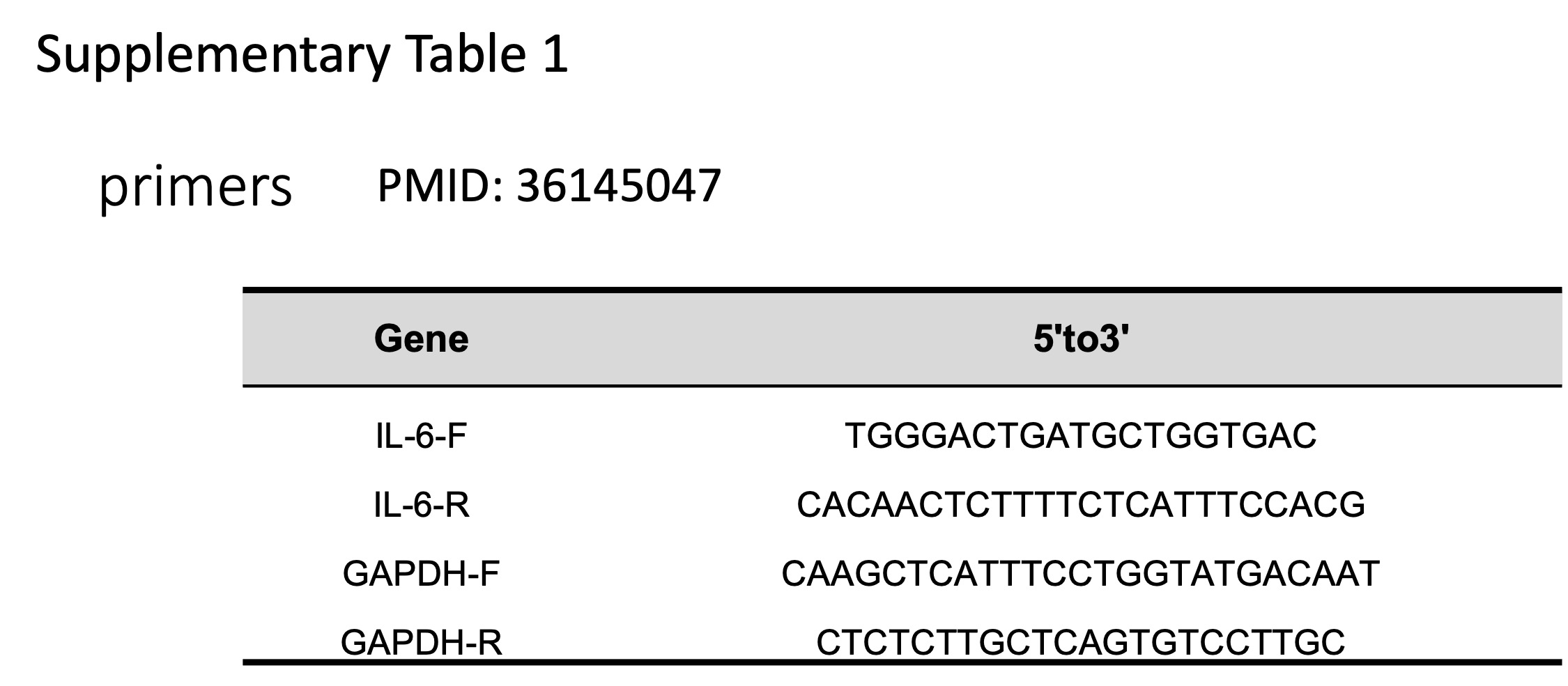

Supplement: Supplementary file 1 [file Supplementary_file_1.jpeg]

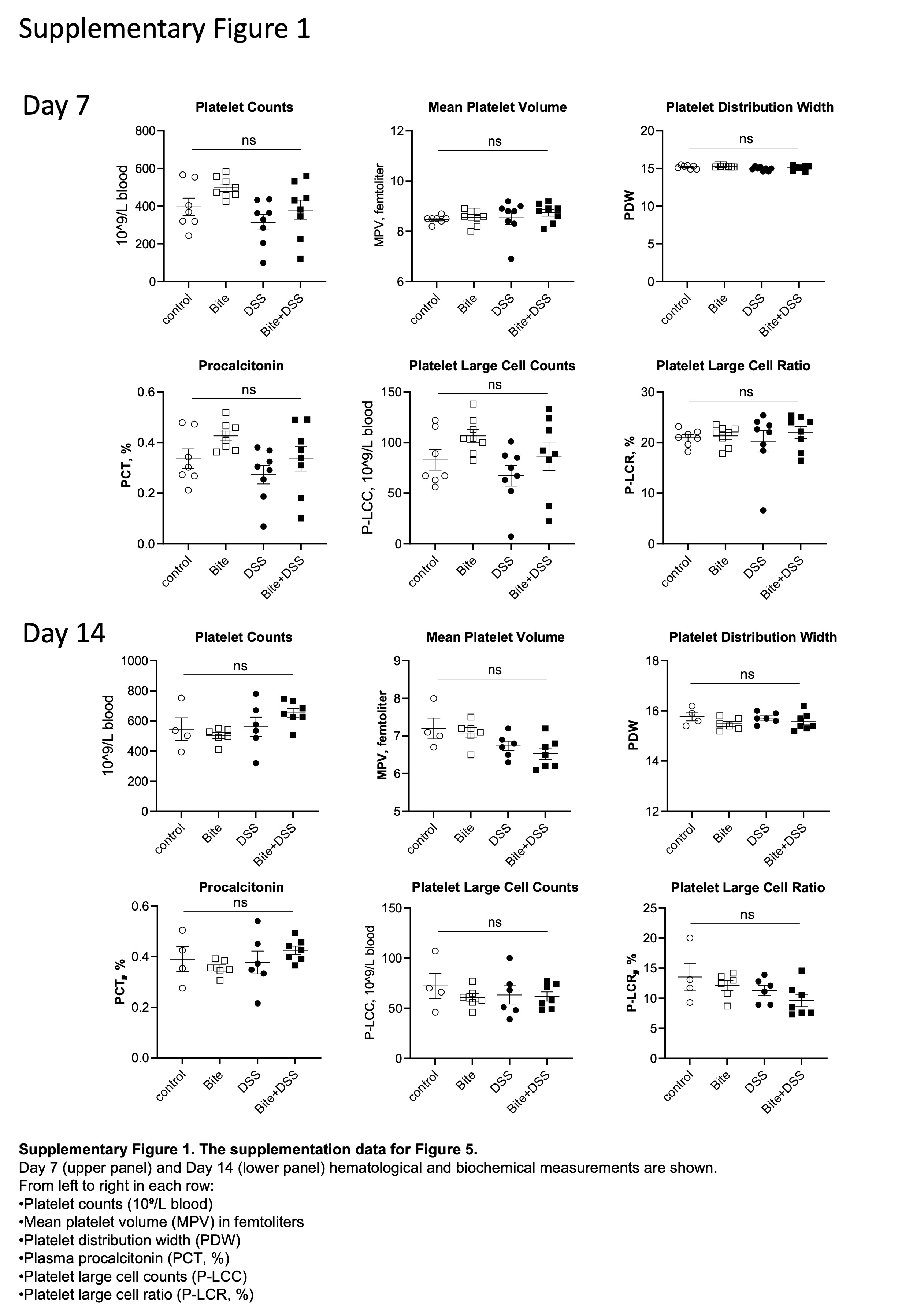

Supplement: Supplementary file 2 [file Supplementary_file_2.jpeg]
